# Treating Woman with Myo-Inositol Vaginal Suppositories Improves Partner's Sperm Motility and Fertility

**DOI:** 10.1155/2016/7621942

**Published:** 2016-06-14

**Authors:** Mario Montanino Oliva, Roberta Poverini, Rosella Lisi, Maria Cristina Carra, Franco Lisi

**Affiliations:** ^1^Department of Obstetrics & Gynecology, Santo Spirito Hospital, 00100 Rome, Italy; ^2^IVF Unit, Villa Mafalda Clinic, 00100 Rome, Italy

## Abstract

Motility is the feature that allows spermatozoa to actively reach and penetrate the female gamete during fertilization. When this function is altered, and especially decreased, troubles in conceiving may occur. In this study, we demonstrated that treating fertile women with myo-inositol (MI) vaginal suppositories ameliorated their partners' sperm motility and also positively affected their conceiving capacity, without changes in cervical mucus structural and biochemical characteristics. Indeed, by means of the postcoital test on female cervical mucus, a significant improvement especially in progressive sperm motility was recorded after MI suppository use. Concomitantly, after MI treatment, a reduction of immotile spermatozoa percentage was observed. Importantly, MI vaginal supplementation positively correlated with a pregnancy for 5 of the 50 couples enrolled in the study, leading us to speculate that this substance may substantially contribute to create in the cervical mucus an ideal* milieu* that makes spermatozoa more motile and functionally able to fertilize. Even though the detailed mechanism is still unclear, these results should encourage MI vaginal use for the clinical improvement of male infertility, through their partners.

## 1. Introduction

Infertility is a medical, psychological, and social problem of couples in reproductive age [1]. Male factor issues play an etiologic role in a substantial portion (10–15%) of couples with conceiving troubles [2].

Different causes may conduct to male infertility: varicocele, cryptorchidism, and hypogonadism are the most frequently observed; on the other hand, in several cases infertility is determined by sperm parameters below the World Health Organization (WHO) reference values [3].

In particular, sperm motility is a critical factor in determining semen quality and fertilizing ability, considered as potential for movement, cervical mucus penetration, capacitation, zona recognition, acrosome reaction, and sperm-oocyte fusion [4–7].

*In vitro* studies evidenced that inositols, and mainly the prevalent isomer myo-inositol (MI), are sugars strongly involved both in spermatozoa maturation and in their migration from the epididymis [8–11], suggesting a potential role for these molecules in affecting sperm motility and fertility.

A recent prospective double-blind randomized placebo controlled study determined that MI improves sperm parameters and serum reproductive hormones in patients with idiopathic infertility [12].

On these findings, we determined if male mild infertility, assessed by spermiogram as reduced sperm motility in respect to WHO criteria, could be improved after treating the fertile female partner with MI vaginal suppositories (Xyminal®; Lo.Li Pharma s.r.l., Rome, Italy).

By the postcoital test on female cervical mucus, we investigated if male sperm motility parameters, especially progressive motility and immotility, could be ameliorated after female MI vaginal use. Indeed, MI may contribute to create a more comfortable environment to facilitate sperm progression and sperm-oocyte cross-talk. The number of couples who ameliorated their fertility after treatment, acquiring conceiving ability, was also registered.

## 2. Materials and Methods

### 2.1. Study Population and Inclusion/Exclusion Criteria

For this study 50 couples who did not achieve a pregnancy after at least 1 year of unprotected intercourse with the same fertile partner were enrolled.

In particular, male patients included showed a mild infertility with troubles in conceiving from 12 to 36 months. Their infertility was assessed by spermiogram according the WHO references and they all fell in the range between the 5th and the 40th percentile, with motility lower than WHO values (40% sperm with progressive, slowly progressive and nonprogressive movement, and 32% sperm with progressive and slow progressive movement) [3].

All female partners were fertile and able to be pregnant.

Men with azoospermia, severe oligozoospermia, or an identifiable cause of infertility were excluded from the study, as well as couples where sterility was due to the female partner.

All the couples were under treatment at the IVF Unit (Villa Mafalda Clinic, Rome, Italy) and they all have signed an Informed Consent Form.

### 2.2. Protocol Design and Treatment

The primary outcome of this study was to investigate if MI vaginal suppositories, given to fertile females, can ameliorate the impaired motility and poor quality of their partners' sperm.

The secondary outcome was to test if MI vaginal suppositories can increase the number of pregnancies in these couples.

This case-control open label study used two-phase strategies, lasting two menstrual cycles. In the first stage, at the 9th day of female menstrual cycle, a baseline spermiogram analyzing sperm parameters was performed on male partners, according to WHO references [3]. Sperm motility (% of total motility, progressive motility, not progressive motility, and static spermatozoa) was assessed. Concomitantly, between the 9th and the 11th day of the menstrual cycle, ultrasound monitoring of female ovulation was followed, and the day after leading follicle was >18 mm couple was requested to have intercourse. The postcoital test on female partner was performed 3–6 hours after the intercourse.

In the second stage, in correspondence with the 9th day of female menstrual cycle, a second spermiogram was performed on male partners and the ultrasound monitoring of female ovulation was followed. When the leading follicle was >16 mm, female partners started the treatment with MI vaginal suppositories (2 mg of MI, Xyminal; Lo.Li Pharma s.r.l., Rome, Italy). The treatment consisted of one vaginal suppository for three consecutive days in the periovulation period (i.e., from the 11th to the 13th one of a regular menstrual cycle). The morning subsequent to the administration of the last suppository, after verifying leading follicle was >18 mm, couples were requested to have the intercourse and 3–6 hours later the postcoital test was performed.

### 2.3. Sperm Analysis

Semen samples were obtained after 3 days of abstinence. They were collected into sterile containers and allowed to liquefy at 37°C for 30 min. A routine sperm analysis was carried out according to WHO criteria [3].

Nonprogressive motility (all other patterns of motility with an absence of progression), progressive motility (spermatozoa moving actively, either linearly or in a large circle, regardless of speed), total motility (nonprogressive motility + progressive motility), and static spermatozoa were determined.

### 2.4. Postcoital Test (PCT)

PCT was performed 3–6 hours after coitus, as described by Hull et al. [13] and according to the WHO procedure [3]. Briefly, we exposed the cervix with a speculum and gently wiped the external os with a cotton swab to remove the external pool of vaginal contaminants. We removed the exocervical mucus with the swab or with forceps and collected cervical mucus from the endocervical canal by aspiration with a tuberculin syringe (without needle).

We advanced the tip of the device approximately 1 cm into the cervical canal before applying suction. Then, we maintained suction as the device was withdrawn. Just before the device was completely withdrawn from the external cervical os, we released the suction pressure.

The evaluation of the cervical mucus properties included the assessment of spinnbarkeit, ferning (crystallization), viscosity, and pH, according to the system devised by Moghissi [14].

Sperm total and progressive and nonprogressive motility were evaluated following WHO criteria [3].

### 2.5. Statistical Analysis

The results are presented as mean ± standard deviation (SD). Differences were statistically analyzed with Student's paired *t*-test. *p* < 0.05 was considered statistically significant.

## 3. Results

For this study 50 couples were enrolled; for each couple, the man was affected by mild infertility while the woman was fertile and able to conceive. Patients' average baseline characteristics are summarized in Table 1.

Spermiogram showed that sperm motility, as well as the other sperm parameters, did not change between the first and second phase of the study, as reported in Table 2.

On the other hand, the postcoital test performed on cervical mucus showed an improvement in sperm total motility, after MI supplementation (Table 3). In particular, sperm progressive motility resulting significantly increased (*p* < 0.001), compared to nonprogressive motility, and the number of immotile spermatozoa decreased as well.

PCT also evidenced that MI supplementation did not influence neither positively nor negatively the examined cervical mucus characteristics; indeed no significant differences were recorded after MI vaginal insertion in respect to the optimal baseline values, as reported in Table 4.

Moreover, as second clinical result, five couples were able to conceive after MI treatment.

Patients were kept under medical check for 30 days after the end of the treatment and no side effect was observed during or after the treatment with MI.

## 4. Discussion

Sperm dysfunction is one of the most commonly observed causes of infertility [15, 16]. Its primary manifestation is poor motility, which negatively impacts on conceiving [17–19]. Italian researchers firstly demonstrated that inositols and, in particular, MI* in vitro* improved sperm parameters, such as motility. Indeed, after incubation with MI, sperm progressive motility increased and the concentration of motile spermatozoa doubled as well, in both normozoospermic men and patients with abnormal sperm parameters [9–11]. Moreover, they observed an amelioration of sperm mitochondrial function that positively correlates with total and progressive motility as well as with sperm quality [11, 20, 21].

On these bases, great attention to the ionic mechanisms and the involvement of protein kinases and phosphatases in regulation of sperm motility was paid. Ca^2+^ was found to be a key regulator, and elevated intracellular Ca^2+^ concentrations were required for both the initiation and maintenance of sperm motility [22–25]. Inositols binding opens calcium channels, increasing ionic intracellular concentrations in the flagellum [26]. Also MI, through PKA-, PKB-, and PKC-dependent pathways, modulates intracellular Ca^2+^ concentrations by acting on the sperm plasma membrane, mitochondria, acrosome, and neck region [27–31]. Thus, Ca^2+^ release is mandatory for all the events that allow the spermatozoa to digest the zona pellucida and penetrate into the oocyte in order to fertilize it [6].

These findings were confirmed by a recent* in vivo* study, in which Calogero and colleagues showed that MI improved sperm parameters, such as motility, and serum reproductive hormones in patients with idiopathic infertility, confirming that MI supplementation should be encouraged in these patients [12].

Following these important clinical results, our aim was to investigate if men with mild infertility could benefit from the treatment of their fertile female partner with MI vaginal suppositories.

Indeed, on a total of 50 couples we intended preliminarily to understand if a supplemented vaginal environment could ameliorate sperm quality and positively modulate sperm motility parameters and their conceiving ability.

To address this issue, two postcoital tests (PCT) were performed in the two stages of the trial and results were compared showing interesting difference after MI vaginal use in terms of spermatozoa motility, evaluated as total, progressive, and nonprogressive motility and as immotility percentage.

The aims of a PCT are to determine the number of active spermatozoa in the cervical mucus and to evaluate sperm survival [32] and sperm behavior some hours after coitus (the reservoir role of mucus) [14].

Anyway, the use of PCT in the basic fertility workup has been subject to debate for so long [33–37]. The conclusion was that this test is an effective predictor of conception, if defined female causes of infertility are absent and duration of infertility is less than 3 years [38].

In particular, our PCT recorded that progressive motility greatly increased and, concomitantly, the number of immotile spermatozoa decreased after MI vaginal supplementation to women. These data did not depend on changes in their cervical mucus structural and biochemical features after MI treatment. In fact, these parameters resulted as being quite unmodified in respect to baseline values. Therefore, we hypothesized that the interaction between spermatozoa and cervical mucus microenvironment could be responsible for the amelioration in sperm motility, determining the ideal conditions to trigger those mechanisms ending with protein kinases activation and intracellular Ca^2+^ release, mentioned above.

As second important result, we evidenced that the treatment has led to a pregnancy for five couples. This interesting result may support the positive correlation MI supplementation-sperm motility-fertility and should be further verified in a larger cohort study.

Despite the intrinsic limitations of the study (i.e., low number of patients which slightly reduced the study's statistical power), overall the data herein reported are encouraging and promote the successful clinical use of MI in women to positively affect their partners' sperm motility and infertility.

Further studies are needed to better clarify* in vivo* the cross-talk between spermatozoa and cervical mucus, in order to understand the mechanisms that MI might trigger and control to improve fertility outcome.

## Figures and Tables

**Table 1:** Patients' baseline average features.

	Age	Weight (Kg)	Height (cm)	BMI
Females (*n* = 50)	34.5 ± 3.6	57.6 ± 4.2	167.3 ± 5.7	23.1 ± 1.8
Males (*n* = 50)	37.1 ± 4.1	78.4 ± 3.9	178.6 ± 6.9	25.2 ± 1.6

Mean ± SD

BMI, body mass index.

**Table 2:** Sperm parameters by spermiogram.

	1st stage	SD	2nd stage	SD	*p* value
Total motility (PR + NP) (%)	30.2	±11.2	30.3	±10.8	NS
PR (%)	15.3	±8.5	15.9	±8.0	NS
NP (%)	14.9	±5.3	14.4	±5.4	NS
IM (%)	69.8	±15.8	69.7	±16.8	NS
Semen volume (mL)	2.72	±0.61	2.5	±0.63	NS
Total sperm number (10^6^ per ejaculate)	72.0	±28.37	70.2	±25.23	NS
Sperm concentration (10^6^ per mL)	25.0	±9.5	23.0	±8.7	NS
Sperm morphology (normal forms, %)	21.0	±20.19	22.0	±20.4	NS

PR = progressive motility; NP = nonprogressive motility; IM = immotility.

**Table 3:** Postcoital test results on sperm parameters.

	Baseline	SD	+Xyminal	SD	*p* value
Total motility (PR + NP) (%)	40.8	±20.4	50.8	±11.1	*p* < 0.05
PR (%)	15.8	±10.6	29.0	±7.5	*p* < 0.001
NP (%)	25.0	±21.2	21.8	±6.7	NS
IM (%)	59.2	±20.4	49.2	±10.5	*p* < 0.05
Sperm number per HPF	3.0	±1.43	3.0	±1.53	NS

PR = progressive motility; NP = nonprogressive motility; IM = immotility; HPF = high-power field.

**Table 4:** Postcoital test results on cervical mucus.

	Baseline	SD	+Xyminal	SD	*p* value
Viscosity	2.7	±0.48	2.9	±0.37	NS
Spinnbarkeit	8.7	±1.05	8.6	±1.01	NS
Ferning	2.6	±0.49	2.6	±0.49	NS
pH	7.55	±0.3	7.3	±0.2	NS
